# Case report: *De novo* mutation of a-galactosidase A in a female patient with end-stage renal disease: report of a case of late diagnosis of Anderson–Fabry disease

**DOI:** 10.3389/fgene.2023.1122893

**Published:** 2023-09-14

**Authors:** Irene Simonetta, Renata Riolo, Federica Todaro, Vincenzo Donadio, Alex Incensi, Salvatore Miceli, Paolo Colomba, Giovanni Duro, Antonino Tuttolomondo

**Affiliations:** ^1^ Internal Medicine and Stroke Care Ward, Regional Reference Center for Diagnosis and Treatment of Anderson-Fabry Disease, Department of Health Promotion, Maternal and Child Health, Internal Medicine and Specialty Excellence “G. D'Alessandro” (PROMISE), University of Palermo, Palermo, Italy; ^2^ Neuromuscular and Neuroimmunology Unit, Bellaria Hospital, IRCCS Institute of Neurological Sciences of Bologna, Bologna, Italy; ^3^ Institute for Biomedical Research and Innovation (IRIB-CNR), National Research Council of Italy, Palermo, Italy

**Keywords:** Anderson–Fabry disease, *de novo* pathogenic variant, one family member, renal disease, case report

## Abstract

**Background:** Anderson–Fabry disease (AFD) is an X-linked disease that results from reduced activity of the enzyme galactosidase alpha (GLA). When the GLA gene sequence is altered by mutations that alter the normal DNA sequence, variants of the alpha-galactosidase A enzyme are produced, which may or may not function. These mutations are responsible for Fabry disease, and to date, over 800 different mutations of the gene have been described in patients with Anderson–Fabry disease. In this case, we report the case of a woman who is the sole family member with this type of mutation.

**Case presentation:** We report a case of a 52-year-old woman with end-stage chronic kidney disease in dialysis treatment. The patient’s alpha-galactosidase activity was 6.6 nmol/ml/h in whole blood, and lyso-GB3 levels were 11.45 nmol/L (normal range < 2.3 nmol/L). Alpha-galactosidase A gene sequence analysis revealed a pathogenic variant of c.947dupT in exon 6, leading to the p. I317NfsTer16 amino acid substitution. The genetic analysis did not detect the same mutation in any of the other screened family members.

**Conclusion:** The international Fabry disease genotype-phenotype database (dbFGP) reports a pathogenic variant c.947dupT in exon 6 that is probably associated with a classical phenotype of Fabry disease. In this case report, we report the case of a woman who is the sole family member with this type of pathogenic variant. Similar situations have not been described in the literature for this pathogenic variant, and it represents an important case of inter- and intrafamilial variability in patients with Fabry disease. The literature shows that *de novo* pathogenic variants are frequently found in the context of Fabry disease.

## Background

Fabry disease (Simonetta et al., 2018; Wu et al., 2017) was initially reported in 1898 and is the second most common lysosomal storage disorder after Gaucher disease (Antonino et al., 2013). AFD is characterised by a congenital error related to the X chromosome, thus altering the catabolism of glycosphingolipids due to the enzyme galactosidase A (Simonetta et al., 2018). The implication is the accumulation of globotriaosylceramide in various tissues and organs, including neurons of the peripheral and central nervous systems, skin, eyes, heart, kidneys, and intestines.

Fabry disease exhibits a wide range of phenotypical variability; in particular, it is possible to distinguish a classical form, characterised by multiorgan involvement and early onset, from atypical variants (milder or late-onset) that may be associated with single organ damage. The classic phenotype is typical of male patients. Patients with traditional FD display various symptoms, such as acroparesthesia, hypohidrosis, angiokeratomas, corneal opacities, cerebrovascular lesions, cardiac disorders, and renal dysfunction (Antonino et al., 2013). Several pathogenic variants have been identified (more than 800), including missense mutations, small deletions/insertions, splice mutations, and large gene rearrangements, and a not-yet-clear role has been attributed to intronic mutations and polymorphisms of single nucleotides (Shabbeer et al., 2002).

The determination of the alpha-Gal A activity in the blood of a heterozygous woman could be normal; this is explained by the Lyon hypothesis of random X chromosome inactivation.

Neurological signs and symptoms of Fabry disease were reported more frequently; cardiac, ocular, gastrointestinal, dermatological, auditory, and renal manifestations were also common.

Among the symptoms concerning the central nervous system, cerebrovascular ischaemic events are most frequent, and they appear at an early age.

Progressive accumulation of GL-3 within the endothelium of intracranial blood vessels is believed to play a primary role in vasculopathy and, consequently, in the risk of ischaemic stroke; however, stroke pathogenesis in Fabry disease requires further detailed investigation (Moore et al., 2007).

Regarding stroke, Rolfs et al. showed a high frequency of AFD in a cohort of cryptogenic stroke patients, which corresponds to approximately 1.2% in young stroke patients (Sims et al., 2009).

Cardiac impairment is characterised by progressive left ventricular hypertrophy, resulting in a clinical presentation similar to hypertrophic cardiomyopathy. Supraventricular and ventricular arrhythmias, such as atrial fibrillation and non-sustained ventricular tachycardia, occur frequently in older patients with severe cardiac hypertrophy and often in the advanced stages of the disease (Frustaci and Chimenti, 2007). Other cardiovascular events include myocardial infarction and significant cardiac procedures (e.g., pacemaker placement, bypass, stent placement, valve replacement, and transplantation).

Renal lesions are found in both hemizygous (male) and heterozygous (female) patients.

Histologically, glycolipid deposits accumulate in podocytes, mesangial cells, and endothelial cells in the kidney. This accumulation is also found in the epithelial cells of the distal tubule and in the vascular cell component (endothelial cells of capillaries, veins, arteries, and vascular smooth muscle). The evolution of kidney disease is represented by segmental and global glomerulosclerosis, tubular atrophy, and interstitial fibrosis. In patients with Fabry disease, renal involvement may be suspected due to the presence of proteinuria in urinalysis and renal failure in blood tests (Alroy et al., 2002).

In this case report, we report the case of a woman who is the sole family member with this type of pathogenic variant. This single pathogenic variant, in only one member of a family, further documents how inter- and intrafamilial transmission variability of Fabry disease is possible.

Early identification of these conditions is essential to promptly start therapy and avoid the onset of organ complications.

## Case presentation

The proband is a 52-year-old woman with an intellectual disability from birth and hearing loss. According to her mother, the cause of the intellectual disability and neurodevelopmental disorder in her daughter was neonatal hypoxic-ischaemic encephalopathy. This pathological condition was responsible for her limited social skills and broken personal relationships.

She has suffered from gastrointestinal manifestations since childhood, complaining about abdominal pain, a tendency towards diarrhoea, and acroparesthesia. Over the past 7 years, she has also suffered from chronic kidney disease, due to which she is undergoing dialysis treatment. In the absence of risk factors for chronic renal disease, after the exclusion of more frequent causes of nephropathy, such as autoimmune and inflammatory disorders, and infective diseases, the patient was referred by her referring physician to our centre for further diagnosis.

Due to the patient’s comorbidities and the premature age of dialysis treatment, it was considered appropriate to perform an analysis of the gene sequence of alpha-galactosidase A. This investigation revealed an alteration in exon 6 with a pathogenic variant c.947dupT, which leads to the substitution of the amino acid p. I317NfsTer16.

In view of the report suggestive of FD, a thorough family history was recorded; however, there were no notable pathologies in the family history, and none of the family members had previously undergone genetic testing. The diagnosis of FD was made on the basis of the alteration of this gene sequence and on the basis of the association of this pathogenic variant with AFD (Mutation Search, 2021). Therefore, this investigation is fundamental and makes the screening of all families efficient, with high diagnostic implications on average over three generations surrounding an index case.

**FIGURE 1:**
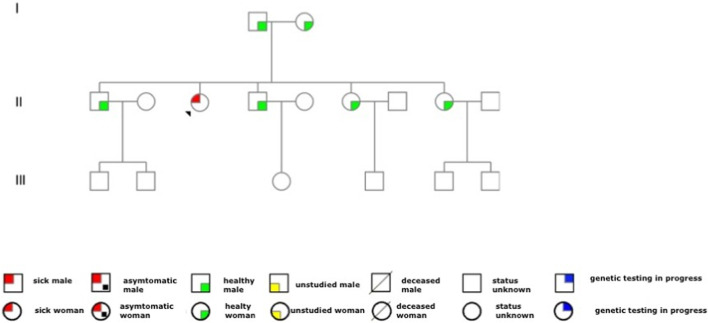
Family pedigree. The red circle with the arrow represents the patient with the disease. As observed from the family tree, the proband is the only member of the family affected by Fabry’s disease. We had the consent of the proband and family members to report the family tree in the case report.

In this case, the genetic analysis did not detect the same mutation in the other family members. The other members of the family were healthy and did not complain of relevant symptoms during the medical examination. The pedigree of the family is shown in Figure 1.

Once the disease is diagnosed, patients must undergo a comprehensive clinical examination and drug treatment.

**FIGURE 2:**
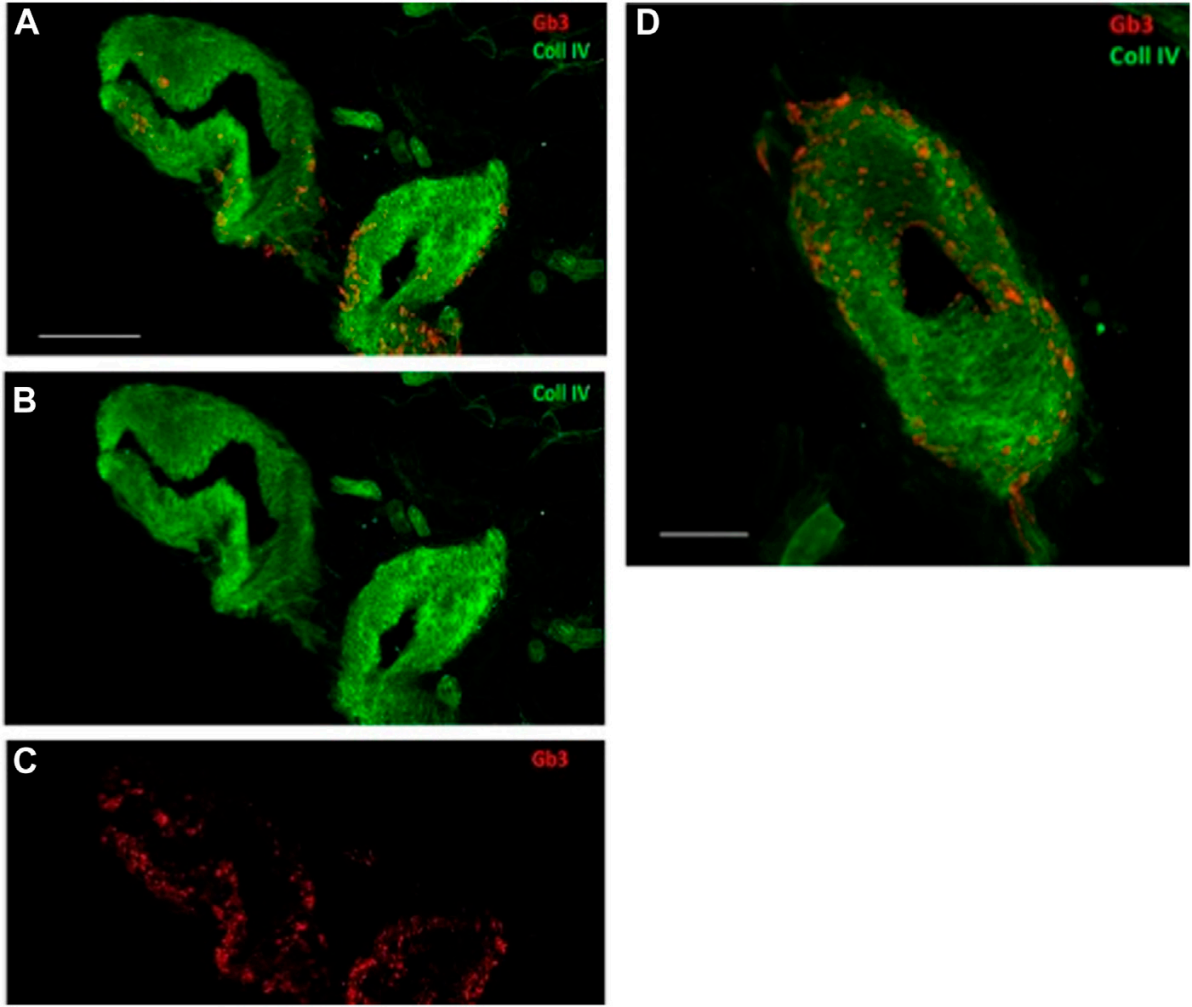
Confocal microscopy (×40 magnification, scale 50 µm) study of Gb3 in skin vessels **(A–C)**. Abnormal deposits were abundant in the walls of the arterioles and their endothelium. **(A–C)** Gb3 deposits and the convergence of the two canals, Gb3 and collagen IV. **(D)** Larger image of the same vessel displaying the merging of the two canals (40X, scale 100 µm).

The patient exhibited 24 h pathological proteinuria in blood chemistry analysis; the electrocardiogram documented sinus rhythm at a heart rate of 72 bpm, left ventricular hypertrophy with overload, and abnormalities of the atriogram. Echocardiography documented a normal-sized left ventricle with concentric hypertrophy. Once this pathology was documented, the patient underwent drug therapy with agalsidase beta. It was considered appropriate to deepen the patient’s diagnostic process by performing a skin biopsy. The aforementioned procedure was performed by taking two skin samples from the leg and thigh, and histological examination showed the presence of neuropathy of the small fibres with predominantly somatic involvement, with positivity for Gb3. These observations are in line with those reported by Liguori et al. (2017) and Liguori et al. (2010). Table 1 reports signs and symptoms that were collected from the prediagnostic phase to the last follow-up visit. Figure 2 reports the confocal microscopy study of Gb3 in skin vessels.

**TABLE 1:** Clinical and laboratory features collected from the prediagnostic phase to the last follow-up visit.

	START	VISIT 1	VISIT 2
α *GAL*	Unknown	6,6 nmol/ml/h	
*GLA MUTATION*	Unknown	c.947dupT	c.947dupT
*LYSO GB3*	Unknown	11.45 nmol/L	10.9 nmol/L
*AGE*	15	52	53
*EYE*	-	-	-
*PAIN*	+	+++	++
*GASTROINTESTINAL TRACT*	+	++	+
*EAR*	-	-	-
*BRAIN*	N.A	N.A	N.A
*HEART*	-	+	++
*KIDNEY*	+++	+++	+++
*SKIN*	-	-	-

-GLA: galactosidase alpha; Lyso Gb3: globotriaosylsphingosine; α GAL: alpha-galactosidase A; N.A: not assessable due to congenital cerebral damage; +, mild; ++ moderate; +++, severe; -: absent.

## Therapeutic intervention and follow-up

The patient was administered therapy with agalsidase beta at a dose of 1 mg/kg once every 2 weeks intravenously. Comorbid therapy was optimised by inserting ACE-I and drugs for neuropathic pain, considering the presence of acroparesthesia. In particular, regarding her known small fibre neuropathy, she complained of burning pain, allodynia, needle sensation of the extremities, and hyperaesthesia; sometimes, she also described a fleeting episode of electric shock-like pain. A few months before the diagnosis of Fabry disease, she reported a worsening of symptoms that became severe and persistent, especially during periods of rest and at night. It appears highly plausible that the gastrointestinal symptoms experienced since childhood, including abdominal pain and diarrhoea, were related to autonomic and enteric dysfunction.

She was subsequently treated with tricyclic antidepressants, amitriptyline, and serotonin–norepinephrine reuptake inhibitors; gabapentin was also added.

The patient is no longer under the care of our reference centre as she died last September.

## Diagnostic assessment

For diagnosis, a dried blood filter paper (DBFP) was used to measure alpha-galactosidase A activity in all family members (Chamoles et al., 2001; Rozenfeld et al., 2006).

Blood samples in EDTA tubes were used for the genetic analysis. Gene amplification was performed through PCR and sequencing; enzyme systems found in exon 6 revealed a pathogenic variant c.947dupT (Chamoles et al., 2001; Rozenfeld et al., 2006; Lukas et al., 2013). This pathogenic variant was detected only in the blood of the patient with severe renal failure, deserving dialysis treatment, and has not been found in any other member of her family.

### Alpha-galactosidase A activity

The enzyme activity found in the blood was approximately 6.6 nmol/ml/h. Normally, the value range is between 0 and 14 nmol/h/mL. The values that are usually found in hemizygotes of Fabry disease range from 0 to 1.5 nmol/h/mL, while for healthy men and women, they are between 3 and 14 nmol/h/mL (Lukas et al., 2013).

### Alpha-galactosidase A pathogenic variant

Through the gene sequence, a c.947dupT pathogenic variant was found in exon 6 due to the amino acid substitution of p. I317NfsTer16 (Lukas et al., 2013).

## Discussion

Through gene sequence analysis, a variety of molecular lesions that can cause FD have been identified, most of which have resulted in point pathogenic variants (Schiffmann et al., 2017).

With regard to heterozygotes, it should be noted that some heterozygous women with a normal enzymatic value may manifest the disease, with a consequent impairment of the quality of life (Schiffmann et al., 2017). This phenomenon is caused by random inactivation of the X chromosome, according to the Lyon hypothesis. Additionally, female subjects with important clinical manifestations could have normal blood values of alpha-galactosidase A; therefore, genetic analysis is necessary for the diagnosis of AFD (Echevarria et al., 2016).

Therefore, in heterozygous female subjects, the enzyme values are not very useful because there is no specific correlation between the degree of enzymatic activity and the clinical presentation of the disease in women; in male subjects, on the other hand, the test is fundamental because it prematurely identifies the sick subjects, documenting a reduction in enzymatic activity, and is useful for the monitoring of therapeutic response (Schiffmann et al., 2017; Echevarria et al., 2016).

The identification of patients to date is of fundamental importance, and it is advisable to identify them from birth to prematurely implement the therapy and avoid the early onset of organ damage or episodes of acute and fatal cerebrovascular cardio-events. Incorrect mutation analysis can lead to an inadequate use of enzyme replacement therapy (ERT) (Echevarria et al., 2016).

The diagnostic approach to this patient was based on detailed analysis and revision of previous laboratory and instrumental reports together with all the anamnestic data collected and, finally, on our diagnostic process with the involvement of a multidisciplinary team (nephrologist, neurologist, geneticist, and pathologist). However, the main limitation of this approach stemmed from challenges in accessing medical assessments performed during the patient’s childhood and adolescence.

The detection of a *de novo* mutation in just one family member is a rare occurrence that highlights the importance of genetic counselling, early diagnosis, and, finally, extended genetic screening for Fabry disease in patients affected by renal disease of unknown aetiology.

## Data Availability

The original contributions presented in the study are included in the article/Supplementary Materials; further inquiries can be directed to the corresponding author.

## Abbreviations

AFD, Anderson–Fabry disease; GB3, globotriaosylceramide; dbFGP, international Fabry disease genotype–phenotype database; TIAs, transient ischaemic attacks; CPB, citrate phosphate buffer.
