# Corrigendum: Case report: *De novo* mutation of a-galactosidase A in a female patient with end-stage renal disease: report of a case of late diagnosis of Anderson–Fabry disease

**DOI:** 10.3389/fgene.2024.1368953

**Published:** 2024-02-15

**Authors:** Irene Simonetta, Renata Riolo, Federica Todaro, Vincenzo Donadio, Alex Incensi, Salvatore Miceli, Paolo Colomba, Giovanni Duro, Antonino Tuttolomondo

**Affiliations:** ^1^ Internal Medicine and Stroke Care Ward, Regional Reference Center for Diagnosis and Treatment of Anderson-Fabry Disease, Department of Health Promotion, Maternal and Child Health, Internal Medicine and Specialty Excellence “G. D’Alessandro” (PROMISE), University of Palermo, Palermo, Italy; ^2^ Neuromuscular and Neuroimmunology Unit, Bellaria Hospital, IRCCS Institute of Neurological Sciences of Bologna, Bologna, Italy; ^3^ Institute for Biomedical Research and Innovation (IRIB-CNR), National Research Council of Italy, Palermo, Italy

**Keywords:** Anderson–Fabry disease, *de novo* pathogenic variant, one family member, renal disease, case report

In the published article, there was an error in the Author list, and authors Salvatore Miceli, Paolo Colomba, Giovanni Duro were erroneously excluded. The corrected Author list appears below.

“Irene Simonetta, Renata Riolo, Federica Todaro, Vincenzo Donadio, Alex Incensi, Salvatore Miceli, Paolo Colomba, Giovanni Duro, Antonino Tuttolomondo.”

In the published article, there was an error in affiliation 1. Instead of “Complex Operating Unit of Internal Medicine with Stroke Care, Regional Reference Center for Diagnosis and Treatment of Anderson-Fabry Disease, Department of Health Promotion, Maternal and Child Health, Internal Medicine and Specialty Excellence “G. D’Alessandro” (PROMISE), University of Palermo, Palermo, Italy”, it should be “Internal Medicine and Stroke Care Ward, Regional Reference Center for Diagnosis and Treatment of Anderson-Fabry Disease, Department of Health Promotion, Maternal and Child Health, Internal Medicine and Specialty Excellence “G. D’Alessandro” (PROMISE), University of Palermo, Palermo, Italy”.

The authors apologize for this error and state that this does not change the scientific conclusions of the article in any way. The original article has been updated.

